# Changes in Lacto-Fermented *Agaricus bisporus* (White and Brown Varieties) Mushroom Characteristics, including Biogenic Amine and Volatile Compound Formation

**DOI:** 10.3390/foods12132441

**Published:** 2023-06-21

**Authors:** Elena Bartkiene, Paulina Zarovaite, Vytaute Starkute, Ernestas Mockus, Egle Zokaityte, Gintare Zokaityte, João Miguel Rocha, Romas Ruibys, Dovile Klupsaite

**Affiliations:** 1 Department of Food Safety and Quality, Veterinary Academy, Lithuanian University of Health Sciences, LT-47181 Kaunas, Lithuania; elena.bartkiene@lsmuni.lt (E.B.); paulina.zarovaite@stud.lsmu.lt (P.Z.); vytaute.starkute@lsmuni.lt (V.S.); 2 Institute of Animal Rearing Technologies, Faculty of Animal Sciences, Lithuanian University of Health Sciences, LT-47181 Kaunas, Lithuania; ernestas.mockus@lsmuni.lt (E.M.); egle.zokaityte@lsmuni.lt (E.Z.); gintare.zokaityte@lsmuni.lt (G.Z.); 3 CBQF—Centro de Biotecnologia e Química Fina—Laboratório Associado, Escola Superior de Biotecnologia, Universidade Católica Portuguesa, 4169-005 Porto, Portugal; jmfrocha@fc.up.pt; 4 LEPABE—Laboratory for Process Engineering, Environment, Biotechnology and Energy, Faculty of Engineering, University of Porto, 4200-465 Porto, Portugal; 5 ALiCE—Associate Laboratory in Chemical Engineering, Faculty of Engineering, University of Porto, 4200-465 Porto, Portugal; 6 Institute of Agricultural and Food Sciences, Agriculture Academy, Vytautas Magnus University, LT-44244 Kaunas, Lithuania; romas.ruibys@vdu.lt

**Keywords:** button mushrooms, lactic acid bacteria, fermentation, aroma compounds, fatty acids, emotions

## Abstract

This study aimed to evaluate the changes in *Agaricus bisporus* (white and brown) characteristics (colour and acidity parameters, lactic acid bacteria (LAB) and mould/yeast counts, biogenic amine content, fatty acid (FA) and volatile compound (VC) profiles, overall acceptability, and emotions induced for consumers) during a 48 h lactic acid fermentation with *Lacticaseibacillus casei* No. 210, *Lactiplantibacillus plantarum* No. 135, *Lacticaseibacillus paracasei* No. 244, and *Pediococcus acidilactici* No. 29 strains. Fermented white and brown *A. bisporus* showed higher LAB count and lower pH, lightness, redness, and yellowness than non-fermented ones. Yeast and fungi counts were similar between non-fermented and fermented samples. All samples contained spermidine (on average, 191.5 mg/kg) and some of the fermented samples had tyramine (on average, 80.7 mg/kg). Saturated FA was the highest in non-fermented brown *A. bisporus*. The highest monounsaturated and polyunsaturated FA contents were found in *Lp. plantarum* No. 135 fermented white and brown *A. bisporus*, respectively. For the first time, the VC profile of fermented *A. bisporus* was analysed. 1-Octen-3-ol content significantly decreased while benzyl alcohol, acetoin, and 2,3-butanediol increased in most fermented samples. Fermented *A. bisporus* received good acceptability scores. The emotional evaluation showed that the LAB strain and the interaction of the LAB strain and *A. bisporus* variety were significant on the intensity of emotions “happy” and “sad”, while all analysed factors and their interactions were significant on the intensity of “angry” and “disgusted” (*p* ≤ 0.05). The findings of this study show the potential of the selected LAB strains and contribute to the increasing body of research on fermented mushrooms.

## 1. Introduction

Edible mushrooms have been regarded as nutritional food, nutraceuticals, pharmaceuticals, and food flavour agents due to their rich chemical composition and health benefits [1]. *Agaricus bisporus* is the most prevalent commercially grown mushroom in the entire world and accounts for 35–45% of the global mushroom market, with Europe and North America being the primary producers [2]. Due to differences in cultivation substrates, phase of growth, and pre- and post-harvest circumstances, the nutrient profile of *A. bisporus* can vary [3]. *A. bisporus* consists of a high proportion of moisture and is high in protein (11.0–29.1%) and carbohydrate (51.1%) [4]. These mushrooms contain nine essential amino acids and are a source of non-starch polysaccharides (β-glucans, chitin, and mannans), oligosaccharides, minerals (K, P, Ca, Na, Zn, Mg, Fe, etc.), and vitamins D, C, B1, B2, and B3 [5]. Although *A. bisporus* has a low percentage of fat, it does contain some essential fatty acids including linoleic acid [4]. *A. bisporus* contains biologically active substances that have been proven to have anticancer, antioxidant, antidiabetic, antibacterial, and anti-inflammatory properties [4].

Immature *A. bisporus* with a white or brown cap (chestnut mushroom) and mature *A. bisporus*, so-called Portobello mushrooms, are supplied to the global market [3]. The popularity of brown *A. bisporus* is growing since most customers believe they taste better [6]. However, this type of *A. bisporus* is more susceptible to enzymatic browning during storage [6]. Because *A. bisporus* lacks a protective cuticle layer but has a high protein, moisture, respiration rate, and enzymatic activity, these mushrooms are difficult to keep stable during storage [7]. Various thermal (drying, cooling), chemical (coating, ozone, washing with antimicrobial agents), and physical (packaging, irradiation, ultrasound) preservation techniques are used to improve the storage stability of fresh *A. bisporus*, but they all have their limitations [3]. As processed food, mushrooms are usually sterilized, cooked, marinated, or fried. These treatments can have a considerable impact on the nutritional value (e.g., improved protein digestibility) and physical qualities of mushrooms, as well as an adverse effect on their bioactive compounds content (e.g., loss of vitamins, antioxidants, and other nutrients) [8].

Fermentation is one of the most ancient, time- and cost-effective ways of preserving food [9]. It was reported that lactic acid bacteria (LAB) are used for the production of fermented vegetables and juices (from fruits and vegetables), and this type of functional food is quite attractive for vegetarians and everyone who is seeking a healthy lifestyle [10,11]. Lactic acid fermentation of mushrooms is prevalent in Eastern European countries and Southeast Asia, but it is not widely employed on an industrial basis [12,13]. This method could be a sustainable biological way to not only preserve but also improve the functionality of edible mushrooms. LAB are safe microorganisms (labelled with GRAS status by the US Food and Drug Administration) and can possess antibacterial and antifungal activities, degrade anti-nutritional factors, improve digestibility and flavour of the fermented product, and enrich it with new volatile and bioactive (vitamins, peptides, exopolysaccharides, enzymes and etc.) compounds [14]. Moreover, each LAB strain may possess different technological, functional, and beneficial properties, which affect the attributes and safety of the final fermented product [15]. LAB are employed in a wide range of commercial food fermentations around the world [9]. *Lactiplantibacillus plantarum* is commonly employed in the food industry considering its ability to enhance antioxidant activity and to improve the flavour of food [16]. *Lacticaseibacillus casei* and *Lacticaseibacillus paracasei* are two of the most studied and used probiotic bacteria [17], while *Pediococcus acidilactici* is a potent producer of bacteriocins as well as a potential probiotic strain, used for the improvement of flavour and texture [18]. In contrast to spontaneous fermentation, the application of starter cultures prevents the proliferation of undesirable microorganisms, shortens the duration of the process, and simplifies product standardization [19]. However, attention should be paid to biogenic amine formation in fermented food because these compounds can be produced by microorganisms, including LAB, and, in high concentration, may elicit adverse effects on human health [20].

The changes in various mushroom characteristics during fermentation with LAB have been rarely reported. In the study of Jabłońska-Ryś et al. [19], changes in sugars and organic acid profile were found during *A. bisporus* fermentation with *Lp. plantarum* 299v and *Lp. plantarum* EK3, while the latter strain improved the organoleptic properties of fermented mushrooms. The same LAB strains were used in Jabłońska-Ryś et al. [21]’s studies for the fermentation of white and brown button mushrooms, where authors examined the colour, texture, and biogenic amine content. Skrzypczak et al. [13] used spontaneous fermentation of white button *A. bisporus* for LAB isolation. Radzki et al. [22] reported that *Lp. plantarum* fermentation of *Pleurotus ostreatus* mushroom polysaccharide resulted in considerable modifications in its chemical composition.

This study aimed to evaluate the changes in *A. bisporus* (white and brown varieties) mushroom characteristics (colour and acidity parameters, LAB and mould/yeast counts, biogenic amine content, fatty acid and volatile compound profiles, overall acceptability, and emotions induced for consumers) during the lactic acid fermentation with *Lc. casei* No. 210, *Lp. plantarum* No. 135, *Lc. paracasei* No. 244, and *P. acidilactici* No. 29 strains.

Most of the studies about fermented mushroom composition report changes in the main mushroom parameters without paying attention to the possible formation of undesirable compounds. Despite the fact that LAB has a GRAS status and lactic acid fermentation is an acceptable bio-preservation method for various foods, in this study, we would like to pay attention to the fact that some undesirable compounds can also be formed during this process. The main novelty of this study is based on the complex analysis, which includes both desirable and undesirable changes in the mushroom samples during fermentation. Additionally, as far as we know, No. 210, No. 135, No. 244, and No. 29 strains, previously isolated from spontaneously fermented cereals, were applied for the first time for *A. bisporus* mushroom fermentation. Taking into consideration that studies on biogenic amines in mushrooms are scarce and reported data on these compounds significantly differ among species, this study will lead to broader knowledge about the changes in edible mushrooms during lactic acid fermentation.

## 2. Materials and Methods

### 2.1. Edible Mushrooms and Lactic Acid Bacteria Strains Used for Fermentation

*A. bisporus* (white and brown varieties) mushrooms were obtained from the local supermarket in Kaunas (Lithuania). Fresh fruiting bodies were cleaned and boiled in water (200 g mushrooms in 500 mL of water) for 10 min with 6 g of salt. After boiling, mushrooms were cooled to room temperature (22 ± 2 °C) and used for fermentation. Non-fermented boiled mushrooms were analysed as control. 

**Figure 1:**
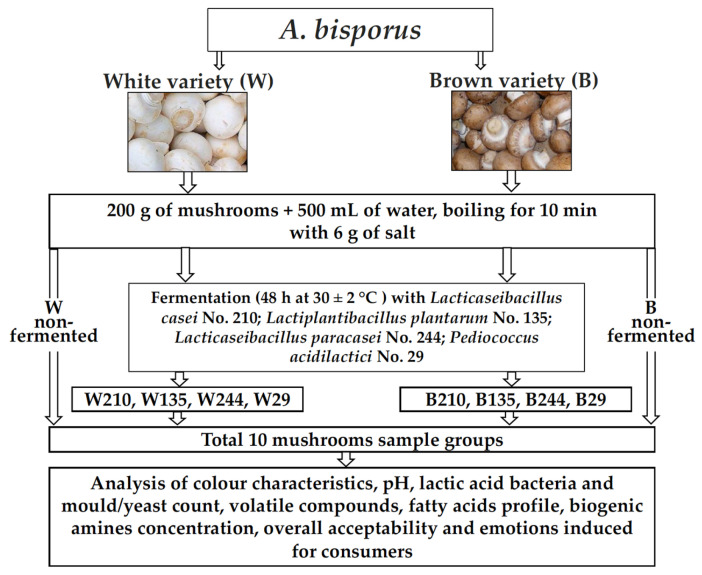
Principal scheme of the experiment (W—*A. bisporus* white variety; B—*A. bisporus* brown variety; non-f—non-fermented samples; 210—fermented with *Lc. casei* No. 210 strain; 135—fermented with *Lp. plantarum* No. 135 strain; 244—fermented with *Lc. paracasei* No. 244 strain; 29—fermented with *P. acidilactici* No. 29 strain).

The LAB strains used in this study were previously isolated from spontaneously fermented cereal and showed a broad spectrum of antimicrobial and antifungal properties [23]. *Lc. casei* No. 210, *Lp. plantarum* No. 135, *Lc. paracasei* No. 244, and *P. acidilactici* No. 29 strains were multiplied in De Man, Rogosa, and Sharpe (MRS) broth culture medium (Biolife, Milan, Italy) at 30 °C under anaerobic conditions. Boiled mushrooms (200 g) were inoculated with 5 mL of LAB multiplied in MRS (average cell concentration 9.0 log_10_ CFU/mL) followed by fermentation for 48 h at 30 ± 2 °C. The principal scheme of the experiment is shown in Figure 1.

### 2.2. Analysis of the Mushrooms’ Colour and Acidity Characteristics, Lactic Acid Bacteria, and Mould/Yeast Counts

Before measuring the mushrooms’ colour characteristics and pH, samples were homogenized using a blender.

The colour coordinates (L*, a*, b*) were assessed using a CIELAB system (Chromameter CR-410, Konica Minolta, Tokyo, Japan) [24].

The pH was measured using a pH electrode (PP-15, Sartorius, Goettingen, Germany) [19].

The total titratable acidity (TTA, °N) was measured according to ISO 750:1998 standard [25]. TTA was determined for a 10 g of mushroom sample homogenized with 90 mL of distilled water and expressed as the volume, in mL, of 0.1 mol/L NaOH required to achieve a pH of 8.2.

The evaluation of the LAB count was performed according to ISO 15214:1998 standard [26]. 10 g of sample was homogenised with 90 mL of aqueous saline (9 g/L NaCl solution). Serial dilutions of 10^−4^ to 10^−8^ with the same saline were prepared for inoculation. Sterile MRS (Man, Rogosa, Sharpe) agar (CM0361, Oxoid, Basingstoke, UK) of 5 mm thickness was used for bacterial growth in Petri dishes. The dishes were separately seeded with the sample suspension using surface sowing and were incubated under anaerobic conditions at 30 °C for 72 h. All results were expressed in log_10_ of colony-forming units per gram (log_10_ CFU/g). 

Yeasts and fungi count in edible mushroom samples were counted on chloramphenicol agar (CM0549, Oxoid, UK) after incubation at 25 °C for 5 days [27]. All experiments were carried out in triplicate, and the number of microorganisms was expressed in log_10_ CFU/g.

### 2.3. Determination of the Mushrooms’ Volatile Compounds (VC)

The VC in edible mushrooms was analysed by gas chromatography–mass spectrometry (GC-MS) according to Bartkiene et al. [28] with some modifications. A solid phase microextraction (SPME) device with Stableflex™ fibre coated with a 50 µm PDMS-DVB-Carboxen™ layer (Supelco, Bellefonte, PA, USA) was used for analysis. Before the experiment, mushroom samples were homogenized using a blender. For headspace extraction, 1 g of homogenized mushrooms was added to the 20 mL extraction vial which was sealed with polytetrafluoroethylene septa and thermostatted at 60 °C for 15 min. The fibre was then exposed to the headspace of the vial for 10 min and desorbed in the injector liner for 2 min (splitless injection mode). Prepared samples were analysed with a GCMS-QP2010 gas chromatograph (Shimadzu, Japan) and a mass spectrometer. The following conditions were used for analysis: injector temperature 250 °C, ion source temperature 220 °C, interface temperature 280 °C. Helium (99.999% detector purity; AGA, Vilnius, Lithuania) was used as carrier gas at a flow rate of 0.97 mL/min. The Rxi^®^-5MS capillary column (0.25 mm ID, 0.25 μm film thickness, 30 m length; Restek, Centre County, PA, USA) was used for analysis. The temperature gradient was programmed from a start at 35 °C (5 min hold) to 200 °C (10 °C/min) up to 280 °C (25 °C/min) (5 min hold). The VC was identified according to mass spectra libraries (NIST11, NIST11S, FFNSC2).

### 2.4. Analysis of the Mushrooms’ Fatty Acid Profile

The extraction of lipids for fatty acids (FA) analysis was performed with chloroform/methanol (2:1 *v*/*v*), and FA methyl esters (FAME) were prepared according to Pérez-Palacios et al. [29] with some modifications. The FA composition of samples was identified using a gas chromatograph GC-2010 Plus (Shimadzu Europa GmbH, Duisburg, Germany) equipped with Mass Spectrometer GCMS-QP2010 (Shimadzu Europa GmbH, Duisburg, Germany). Separation was carried out on a Stabilwax-MS column (30 m length, 0.25 mmID, and 0.25 μm df) (Restek Corporation, Bellefonte, PA, USA). Oven temperature program started at 50 °C, then increased at a rate of 8 °C/min to 220 °C, held for 1 min at 220 °C, increased again at a rate of 20 °C/min to 240 °C and, finally, held throughout 10 min. The injector temperature was 240 °C, the interface −240 °C, and the ion source 240 °C. The carrier gas was helium at a flow rate of 0.91 mL/min. The individual FAME peaks were identified by comparing their retention times with FAME standards (Merck & Co., Inc., Kenilworth, NJ, USA).

### 2.5. Analysis of Biogenic Amine Content in Mushrooms

Sample preparation and determination of biogenic amines (BA) in mushroom samples were performed according to the method of Ben-Gigirey et al. [30], with some modifications described by Bartkiene et al. [31]. Before the experiment, mushroom samples were homogenized using a blender. The following BA were analysed: tryptamine, phenylethylamine, cadaverine, putrescine, histamine, tyramine, spermine, and spermidine. The standard BA solutions were prepared by dissolving known amounts of each BA (including internal standard—1.7-diamino-heptane) in 20 mL of deionised water. Briefly, 5 g of sample were extracted with 10 mL of perchloric acid (0.4 mol/L) twice. The derivatization of sample extracts and standards was performed using a dansyl chloride solution in acetonitrile (10 mg/mL) as a reagent. A Varian ProStar HPLC system (Varian Corp., Palo Alto, CA, USA) equipped with a ProStar 325 UV/VIS Detector and Galaxy software (Agilent, Santa Clara, CA, USA) was used for analysis. A Discovery^®^ HS C18 column (150 × 4.6 mm, 5 µm; SupelcoTM Analytical, Bellefonte, PA, USA) was used to separate BA. Ammonium acetate (0.1 mol/L) and acetonitrile were used as the mobile phases at a flow rate of 0.8 mL/min. The sample volume injected was 20 µL and the amines were monitored at 254 nm. The BA were identified based on their retention times in comparison to their corresponding standards.

### 2.6. Evaluation of the Overall Acceptability and Emotions Induced for Consumers by the Edible Mushrooms

The overall acceptability of the mushroom samples was evaluated by 10 trained judges (from 30 to 50 years old, 7 females and 3 males), according to the International Standards Organization method 8586-1 [32], using a 10-point scale ranging from 0 (‘dislike extremely’) to 10 (‘like extremely’). Samples were also tested by applying FaceReader 8.0 software (Noldus Information Technology, Wageningen, The Netherlands), scaling eight emotion patterns (“neutral”, “happy”, “sad”, “angry”, “surprised”, “scared”, “disgusted”, “contempt”) according to the procedure described by Bartkiene et al. [28]. Each emotion’s intensity was represented on a scale of 0 (no emotion) to 1 (highest intensity of emotion). The valence was calculated as the intensity of “happy” minus the intensity of the negative emotion with the highest intensity. The valence scale ran from −1 to 1.

### 2.7. Statistical Analysis

Results of the microbiological and physicochemical analyses were expressed as the mean (*n* = 9) ± standard error (SE). Fermentation of mushrooms was performed once by fermenting three parallel samples; all analyses of the parallel samples were performed in triplicate. Results of the overall acceptability and emotions induced for judges by the edible mushrooms were expressed as the mean (*n* = 10) ± standard error (SE). Results were analysed using the statistical package SPSS for Windows V15.0 (SPSS Inc., Chicago, IL, USA, 2007). A linear Pearson’s correlation was used to quantify the strength of the relationship between the variables. Results were recognized as statistically significant at *p* ≤ 0.05.

## 3. Results and Discussion

### 3.1. Mushrooms’ Colour Characteristics, pH, Lactic Acid Bacteria, and Mould/Yeast Count

Colour characteristics and acidity parameters of the non-fermented and fermented mushroom samples are shown in Table 1. In comparison to non-fermented samples’ colour coordinates, the white variety of *A. bisporus* showed higher L* and b* coordinates (on average, by 14.7 and 3.24%, respectively), but lower a* coordinates (on average, by 20.5%), in comparison with brown-variety samples. In all cases (both white and brown varieties of mushroom), fermented samples showed lower L*, a*, and b* coordinates, in comparison with non-fermented ones. Multivariate analysis of variance showed that the variety of mushrooms is a significant factor in samples’ a* coordinates (*p* = 0.005). However, the LAB used for fermentation and mushroom variety, and the interaction of these factors were not significant on L* and b* coordinates. 

This study’s results confirmed the data published by other authors, which indicate that lactic fermentation influences the colour parameters of mushrooms and reduces the lightness of mushroom fruiting bodies [12]. During lactic fermentation, also, significant changes in the a* and b* colour coordinates can be obtained [33]. LAB can produce a variety of metabolites, including various organic acids, short-chain fatty acids, amines, bacteriocins, vitamins, exopolysaccharides, etc. [34]. Additionally, LAB excretes a variety of enzymes [35], which are involved in fermentable substrate modification. Finally, these complex mechanisms can also be involved in the degradation of coloured compounds. It was reported that the decrease in the L* coordinate could be related to enzymatic and non-enzymatic browning in mushrooms [36].

Comparing the samples’ acidity parameters, the lowest pH was found in samples fermented with the *Lc. paracasei* No. 244 strain (white variety-sample pH was 4.24, brown-variety sample pH was 4.33). However, the analysed factors (variety of mushrooms and LAB strain used for fermentation) and their interactions were not significant on sample pH. The highest TTA was observed in white mushroom samples fermented with *Lc. casei* No. 210 and *Lc. paracasei* No. 244 strains (0.40° N). Despite the fact that a correlation between samples pH and TTA was not established, moderate positive correlations between samples TTA with L* and b* coordinates were found (r = 0.612, *p* ≤ 0.001 and r = 0.497, *p* = 0.005). 

Fresh mushrooms’ pH is neutral [37]. The pH decrease during the fermentation process is associated with the accumulation of organic acids [38,39]. As well, the organic acid production of different microorganisms, including LAB strains, varies according to their characteristics [40]. The mushroom’s fruiting body can consist of 35 to 70% carbohydrates [41]. It was reported that LABs are able to ferment most of the mushroom carbohydrates, and lactic fermentation of mushroom fruiting bodies is possible without the addition of sugar [12]. Fungal carbohydrates include monosaccharides (i.e., glucose) and disaccharides (i.e., trehalose), sugar alcohols (i.e., mannitol), polysaccharides, glycogen, and chitin [42]. The use of selected LAB strains for mushroom fermentation ensures a correct process [12]. This study showed that the lowest pH of the mushrooms can be obtained by applying, for their fermentation, the *Lc. paracasei* No. 244 strain, which, in our previous studies, showed characteristics to ferment a broad spectrum of carbohydrates [23]. Additionally, the use of selected LAB increases the health-beneficial value of mushrooms due to the presence of viable LAB cells [43]. LAB, yeast, and fungi counts in non-fermented and fermented mushroom samples are shown in Table 2.

In terms of LAB, in yeast and fungi counts in non-fermented mushrooms, significant differences were not established (on average, the LAB count in non-fermented mushrooms was 1.52 log_10_ CFU/g; yeast and fungi count in non-fermented mushrooms was 1.39 log_10_ CFU/g) (Table 2). Additionally, significant differences in non-fermented and fermented samples in yeast and fungi counts were not established; however, fermented samples showed significantly higher LAB counts in comparison with non-fermented ones. The highest LAB count was established in fermentation with *Lc. casei* No. 210 and in with *Lp. plantarum* No. 135 strains in white- and brown-variety mushroom samples (on average, 7.81 and 7.47 log_10_ CFU/g, respectively). The LAB count in samples fermented with *Lc. paracasei* No. 244 and *P. acidilactici* No. 29 strains was, on average, 6.50 log_10_ CFU/g. 

It was reported that many LABs can be applied for mushroom fermentation, including, *Lp. plantarum* [43,44,45,46,47,48,49], *Lactobacillus delbrueckii* subsp. *bulgaricus*, *Levilactobacillus brevis*, *Lc. casei*, *Lactobacillus helveticus*, *Lactiplantibacillus pentosus*, *Streptococcus lactis*, *Lactococcus lactis*, *Leuconostoc mesenteroides*, and *Propionibacterium freudenreichii* [43,44,45,46,50]. Jabłonska-Ry’s et al. [33] reported that *Lp. plantarum* 299v strain contributed to a rapid reduction of the pH value of mushrooms, and the finished products showed a high number of viable LAB cells.

Venturini et al. [37] reported that in fresh mushrooms, the LAB count is lower, at 2.0 log_10_ CFU/g. However, yeast and mould co-existed with LAB in the process of fermentation [12]. The negative activity of yeast in the lactic fermentation process may lead to a decrease in fermentable substrate acidity [12]. However, these processes do not occur at pH 4.5 and below [51]. In our study, correlations between LAB count and acidity parameters, as well as between yeast and fungi count and acidity parameters were not found. However, the strain used for fermentation was a significant factor in LAB count in fermented samples (*p* = 0.004). 

Microbial interactions play an important role in the diversity of microbial communities, which plays a key role in selecting functional microbiota and inhibiting the growth of non-desirable microorganisms. However, little is known about microbial interactions during the process of mushroom fermentation, which makes it difficult to control and manage. Although our previous studies showed that the LAB strains used in this experiment possess antifungal properties against some fungi (previously isolated from cereal), it can be that fungi that are present in mushroom masses are resistant to these LAB metabolites. Further studies are needed to indicate which fungi are predominant in mushrooms and to select the most appropriate LAB for their inhibition.

### 3.2. Biogenic Amine Formation in Fermented Mushroom Samples

Tryptamine, phenylethylamine, putrescine, cadaverine, and spermine were not found in mushroom samples (Table 3). However, in white- and brown-variety mushroom samples fermented with *Lc. casei* No. 210 strain and in white variety mushroom samples fermented with *Lc. plantarum* No. 135 strain, tyramine was formed (on average, tyramine content was 82.7 mg/kg). The main BA in non-fermented and fermented mushroom samples was spermidine. However, the analysed factors were not significant on spermidine content in mushroom samples. 

The amount of protein and the dry mass of fungal fruiting bodies could vary from 20 to 40% [12]. Our previous studies showed that fermentation as well as ultrasonication could increase BA content in wild mushrooms [31]. Additionally, the presence of putrescine and cadaverine in fresh fruiting bodies of fungi was reported [52,53]. In general, BA synthesis is related to the presence of decarboxylating microorganisms in all foods that contain proteins or free amino acids [20]. Usually, high concentrations of BA in non-fermented foods indicate undesired spoilage and microorganism activity. Despite the fact that LABs are generally recognized as safe microorganisms, BA can be formed by LAB decarboxylase in protein-rich fermented foods [54]. Therefore, the presence of slight quantities of BA in fermented foods is expected. In low concentrations, BA acts as neuromodulators, neurotransmitters, and antioxidants and is important for fertility and protein biosynthesis [20]. However, at higher concentrations, BA causes major food poisoning and other health issues such as headaches, allergies, diarrhoea, raised blood pressure, and cell proliferation [54]. The toxicological effects of BA are mainly associated with tyramine and histamine [55]. The concentration and type of BA in fermentable substrates are related to many factors, including amino acid content and composition, technological conditions, etc. [55]. The acidity of the substrate is also an important factor in the formation of BA [20]. In this study, it was found that the LAB strain used for fermentation is a significant factor in tyramine content in mushrooms (*p* ≤ 0.001). Positive moderate correlations were found between TTA and tyramine (r = 0.463, *p* = 0.010), as well as between TTA and spermidine (r = 0.710, *p* ≤ 0.001). 

Certain BA, including tyramine, can be produced by LAB species in different fermented foods [20]. It was reported that spermidine could be formed from spermine and tyramine from tyrosine [56]. The total BA content in food should not exceed 750–900 mg/kg [57]. According to the results obtained in the present study, all tested samples were below this range. Studies on BA in mushrooms are scarce and reported concentrations of these compounds significantly differ among species [58,59,60]. Jabłonska-Ry’s et al. [59] reported that spermidine and putrescine are the most common BA in mushrooms. Reis et al. [60]’s study showed that commercial mushroom species (fresh) could contain spermidine, phenylethylamine, tyramine, and tryptamine. Jabłońska-Ryś et al. [21] only found spermidine, tyramine, and putrescine in button mushrooms fermented with *Lp. plantarum* 299v, while tyramine was not detected in samples fermented with *Lp. plantarum* EK3. Finally, this study showed that it is very important to select appropriate LAB strains for the fermentation of edible mushrooms to ensure their safety in the case of BA formation.

### 3.3. Fatty Acid Profile of the Non-Treated and Fermented Mushrooms 

The fatty acid (FA) content (% of the total fat content) of the mushroom samples is shown in Table 4. The main FA in *A. bisporus* mushrooms was linoleic (C18:2), and the highest C18:2 content was established with *Lp. plantarum* No. 135 strain-fermented brown-variety samples (80.6% of the total fat content). Additionally, a positive moderate correlation between C18:2 and sample TTA was established (r = 0.777, *p* ≤ 0.001). Palmitic acid (C16:0) content in mushroom samples FA profile varied from 11.0 to 13.0% from the total fat content (in with *Lp. plantarum* No. 135 strain fermented brown variety samples and in with *Lc. casei* No. 210 strain fermented white variety samples, respectively). C16:0 also showed a positive moderate correlation with samples TTA (r = 0.781, *p* ≤ 0.001). The highest 9-octadecenoic acid (C18:1) content was found in *Lp. plantarum* No. 135 strain-fermented white-variety samples (10.6% of the total fat content). However, with the same strain-fermented brown-variety samples showed the lowest C18:1 content (3.44% of the total fat content). Between C18:1 content and LAB count in mushrooms, a negative weak correlation was established (r = −0.367, *p* = 0.046). Non-fermented brown-variety *A. bisporus* mushrooms showed the highest palmitoleic (C16:1) and stearic (C18:0) fatty acids content. C18:0 content in samples showed a positive weak correlation with yeast and fungi count (r = 0.372, *p* = 0.043). Eicosanoic (C20:0) and docosanoic (C22:0) FAs were found in 4 out of 10 tested mushroom samples, in non-fermented brown-variety mushrooms, in *Lc. casei* No. 210 strain-fermented white- and brown-variety mushrooms, and in *Lp. plantarum* No. 135 strain fermented brown variety samples. Multivariate analysis of variance showed that mushroom variety was a significant factor in C22:0 content (*p* ≤ 0.001); the LAB strain used for fermentation was a significant factor in C16:1, C20:0, and C22:0 content in mushrooms (*p* = 0.008, *p* = 0.001, *p* ≤ 0.001, respectively), and as the analysed factors’ interactions were significant on C20:0 and C22:0 content in mushrooms (*p* = 0.007 and *p* ≤ 0.001, respectively). 

In comparison to FA profiles of the mushroom samples, the highest saturated fatty acids (SFA) content was found in non-fermented brown variety mushroom samples (17.6% of the total fat content). However, the analysed factors and their interactions were not significant on SFA content in mushrooms. The highest monounsaturated fatty acid (MUFA) content was found in *Lp. plantarum* No. 135 strain-fermented white-variety samples (10.6% of the total fat content). The LAB strain used for fermentation, as well as the analysed factors’ interactions, were significant on MUFA content in mushroom samples (*p* = 0.035 and *p* = 0.030, respectively). The highest polyunsaturated fatty acid (PUFA) content was found in *Lp. plantarum* No. 135 strain-fermented brown-variety samples (80.6% of the total fat content); however, the analysed factors were not significant on PUFA content in mushrooms. Omega-3 FAs were not found in mushrooms. The highest omega-6 content was observed in brown-variety *A. bisporus* fermented with the *Lp. plantarum* No. 135 strain, and the highest omega-9 content was observed in white-variety mushrooms fermented with *Lp. plantarum* No. 135 strain. Mushroom variety was a significant factor in omega-9 content in samples (*p* = 0.001). Positive moderate correlations between samples TTA and SFA, PUFA, and omega-6 were established (r = 0.750, *p* ≤ 0.001; r = 0.777, *p* ≤ 0.001; and r = 0.777, *p* ≤ 0.001, respectively).

It was reported that mushroom lipids consist mainly of MUFAs and PUFAs, which are classified as healthy sources of lipids [61]. Although the crude fats in mushrooms are present only in small amounts, a considerable amount of linoleic acid is present in *A. bisporus* [62]. Stojković et al. [63] reported that *A. bisporus* lipids profile consists of myristic acid (C14:0) (0.58% of the total fat content), C16:0 (14.02% of the total fat content), C16:1 (0.42% of the total fat content), C18:0 (6.54% of the total fat content), C18:1n-9 (20.58% from the total fat content), C18:2n-6 (43.87% of the total fat content), α-linoleic acid (C18:3 n-3) (3.25% of the total fat content), C20:0 (3.16% of the total fat content), and C20:1 n-9 (0.08% of the total fat content). 

According to Saiqa et al., the FA profile of *A. bisporus* comprises stearic acid, palmitic, linoleic, caprylic, oleic, erucic, and eicosanoic acids [64]. Additionally, it was reported that palmitic (12.67–14.71%) and linoleic (61.82–67.29%) FAs are the main FAs in *A. bisporus* [65]. As well, Shao et al. [66] reported that palmitic, linoleic, and stearic acids are the major FAs in *A. bisporus*’s lipid profile. 

However, differences in FA profiles are obtained not just between the species but can also be related to a mushroom’s geographic origin [67]. However, mammals cannot synthesize linoleic and linolenic acids because they lack enzymes for ω-3 desaturation [62]. These FAs are essential and must be present in the human diet [68,69]. It was reported that *A. bisporus* from the Netherlands and Portugal showed the highest levels of omega-3 and -9 [63] and omega-6 [70], respectively. Muszyńska et al. [71] reported that in lyophilized mycelia of *A. bisporus* lipid profile, linoleic FA is predominant (43.9% of the total fat content). Therefore, mushrooms are good sources of essential FA omega-6 [72]. However, a complementary omega-3 source is required for a balanced diet [62]. 

It should be mentioned that the FA profile in cultivated and wild *Agaricus* sp. can vary [73] and the total FA contents can range from 180 to 5818 mg/kg DW. However, linoleic FA represents, on average, 90% of the *A. bisporus* fat content [74]. 

Additionally, the metabolism of LAB can favour lipid oxidation during fermentation or exert strong antioxidative effects [75]. Hydroperoxy linoleic FA is alternatively reduced to hydroxy-linoleic acid with concomitant oxidation of other substrate (in our study, mushrooms) constituents. In the presence of cysteine, peroxides are converted to the corresponding hydroxy-fatty acids [76]. Lactobacilli hydrate oleic, linoleic, and linoleic acids to hydroxyl fatty acids. Linoleic acid is converted to 13-hydroxy-9-octadecenoic acid or the antifungal 10-hydroxy-12-octadecenoic acid [76,77]. The reaction is catalysed by a fatty acid hydratase [78]. The results of this study can be valuable establishing a database about non-treated and fermented *A. bisporus* FA profiles, as well as their changes during fermentation.

### 3.4. Non-Treated and Fermented Mushrooms Volatile Compound Profiles

Volatile compound profiles of the mushroom samples (% of the total volatile compounds) are shown in Table 5. The main volatile compounds in mushroom samples were 1-octen-3-ol and benzyl alcohol. In comparison 1-octen-3-ol content, fermentation significantly reduced this volatile compound content in samples, and in *Lp. plantarum* No. 135 strain-fermented white-variety *A. bisporus*, 1-octen-3-ol was not detected. The LAB strain used for fermentation was a significant factor in 1-octen-3-ol content in mushrooms (*p* = 0.004). In comparing non-fermented white-variety mushrooms with fermented ones, different tendencies in the benzyl alcohol content were established. In white variety samples fermented with *Lc. casei* No. 210 and *Lc. paracasei* No. 244 strains, on average, 1.81 times higher benzyl alcohol content was found. However, in samples fermented with *Lp. plantarum* No. 135 strain, on average, 27.3% lower benzyl alcohol content was established in comparison with non-fermented samples. White-variety samples fermented with the *P. acidilactici* No. 29 strain showed similar benzyl alcohol content compared to non-fermented ones. When comparing non-fermented brown-variety samples with fermented ones, samples fermented with the *Lc. casei* No. 210 strain showed similar benzyl alcohol content compared to non-fermented ones; in samples fermented with the *Lp. plantarum* No. 135 strain, on average, 37.5% lower benzyl alcohol content was established; samples fermented with *Lc. paracasei* No. 244 and *P. acidilactici* No. 29 strains showed, on average, 34.1 and 41.3%, respectively, higher content of benzyl alcohol in comparison with non-fermented samples. The analysed factors and their interactions were not significant in benzyl alcohol content in mushroom samples. 

Acetoin was found in 3 out of 10 analysed samples and the LAB strain used for fermentation was a significant factor in this volatile compound content in mushrooms (*p* = 0.001). 2,3-Butanediol was established in the *Lc. casei* No. 210 strain and in the *Lp. plantarum* No. 135 strain-fermented brown-variety mushrooms, as well as in *Lp. plantarum* No. 135 strain- and in with *P. acidilactici* No. 29 strain-fermented brown variety samples. The analysed factors’ interactions were significant in 2,3-butanediol content in mushrooms (*p* = 0.027). Benzaldehyde was found in most of the mushroom samples, except in *P. acidilactici* No. 29 strain-fermented white-variety samples, and the LAB strain used for fermentation as well as the analysed factors’ interactions were significant in benzaldehyde content in mushroom samples (*p* = 0.001 and *p* = 0.012, respectively). In most of the samples, 3-octanone content was reduced after fermentation, except in *P. acidilactici* No. 29 strain-fermented brown-variety mushrooms. The LAB strain used for fermentation as well as the analysed factors’ interactions were significant in 3-octanone content in mushrooms (*p* ≤ 0.001). Ethylhexanol was found in both non-fermented samples as well as in brown-variety mushrooms fermented with *Lc. casei* No. 210 and *Lp. plantarum* No. 135 strains. All analysed factors and their interactions were significant in ethylhexanol content in samples (*p* ≤ 0.001). Benzeneacetaldehyde was identified in most of the mushroom samples, except in *Lc. paracasei* No. 244-fermented white-variety samples, and mushroom variety as well as the analysed factors’ interactions were significant in benzeneacetaldehyde content in samples (*p* ≤ 0.001 and *p* = 0.002, respectively). In all the cases, a higher content of nonanal was found in fermented samples, and all analysed factors and their interactions were significant in the volatile compound content in mushrooms (mushroom variety *p* = 0.022; LAB strain used for fermentation *p* ≤ 0.001; factors interaction *p* ≤ 0.001). Similar tendencies of dodecane content were established, and in all the cases, a higher content of dodecane was found in fermented samples in comparison with non-fermented ones. The LAB strain used for fermentation and the analysed factors’ interactions were significant on dodecane content in mushrooms (*p* ≤ 0.001 and *p* = 0.004, respectively). Benzo-2,3-pyrrole was found in 3 out of the 10 analysed sample groups, and all analysed factors and their interactions were significant on this volatile compound content in mushrooms (mushrooms variety *p* = 0.050; LAB strain used for fermentation *p* = 0.002; factors interaction *p* = 0.015). In all the cases, fermentation increased tetradecane content in mushrooms’ volatile compounds profiles, and all analysed factors and their interactions were significant in tetradecane content in samples (mushrooms variety *p* ≤ 0.001; LAB strain used for fermentation *p* ≤ 0.001; factors interaction *p* = 0.003). In comparison to 2,4-bis(1,1-dimethylethyl)phenol content in non-fermented and fermented samples, in most of the white-variety samples, this volatile compound content was increased (except samples fermented with *P. acidilactici* No. 29 strain). However, in brown-variety samples, after fermentation with *Lp. plantarum* No. 135 and *P. acidilactici* No. 29 strains, on average, 2.25 and 1.4 times, respectively, lower 2,4-bis(1,1-dimethylethyl)phenol content was found in comparison with non-fermented samples. The LAB strain used for fermentation and the analysed factors’ interactions were significant in 2,4-bis(1,1-dimethylethyl)phenol content in mushrooms (*p* = 0.016 and *p* ≤ 0.001, respectively). 

The volatile compound profile of *A. bisporus* arises from FA and amino acid metabolism and can differ according to growth stages and conditions as well as genotype species [79]. During fermentation, LAB can produce a variety of volatile compounds, thereby giving fermented products a unique odour [16]. To the best of our knowledge, there are no data on the volatile compound profile of fermented *A. bisporus*. Only in the study of Wang et al. [80], the edible mushroom *Pleurotus eryngii* (boiled for 2 min) was fermented with *Lp. plantarum.* Compared to naturally fermented *P. eryngii*, similar contents of alcohols and higher content of aldehydes, ketones, and esters were found in *P. eryngii* fermented with *Lp. plantarum*.

Eight-carbon compound 1-octen-3-ol is a characteristic volatile of button mushrooms that provides a “mushroom-like, earthy, and buttery” odour and is generated during the enzymatic degradation of linoleic FA [81]. A significantly lower amount of this volatile was reported after the thermal treatment of mushrooms [82]. Similar to our study, the diminution of 1-octen-3-ol was also found in oats fermented with *Lc. paracasei* and after fermentation of *Allomyrina dichotoma* larvae with *Lactobacillus acidophillus* and *Lp. plantarum* [83,84].

Amino acid-derived benzyl alcohol, with its aromatic, floral, fruity, and sweet odour, is another dominant volatile in white and brown *A. bisporus*, whose content significantly decreases after heat treatment [5,81,85]. However, in our case, fermentation with LAB increased the content of this volatile, and this is related to the increased formation of alcohols during the process of LAB fermentation [86].

It was reported that the content of such ketones as 3-octanone (mushroom, herbal, lavender note), which is generated via enzymatic oxidation of linoleic or linolenic acids, reduces in *A. bisporus* after heat treatment [81]. Moreover, this volatile can also be degraded by LAB during fermentation [87]. The presence of 2,3-butanediol (fruity, creamy, and buttery notes) and acetoin in some of the fermented white and brown *A. bisporus* samples can be related to the LAB carbohydrate metabolism [88].

The amino acid phenylalanine oxidation metabolites are precursors of benzaldehyde (with the odour of almond, sweet, and phenolic) and benzene acetaldehyde (with the odour of green, sweat, and phenolic) [79]. Dodecane and tetradecane are specific volatiles and were also previously found in *A. bisporus* species [79]. Nonanal (waxy, aldehydic, rose note) and tetradecane (alkane) were previously identified in dried pine mushroom [89]. 2-ethylhexanol was detected in *A. bisporus*, *Volariella volvacea* and *Cortinarius odorifer* mushrooms and provides floral and cedrol woody notes [85,90,91]. 2,4-bis (1,1-dimethylethyl)- phenol was previously found in rare gilled mushrooms of India and Southern Ocean microalgae *Chlorella* sp. PR-1 and possesses biocidal activity [92,93].

### 3.5. Overall Acceptability and Emotions Induced for Judges by the Mushroom Samples

The overall acceptability and emotions induced for judges by mushroom samples are shown in Table 6. Despite that significant differences between mushroom samples’ overall acceptability and emotion “neutral” were not established, other fixed emotions’ intensities varied between the tested sample groups. In the intensity of the “happy” emotion, the highest expression was found with non-fermented white-variety mushroom samples (0.120). The lowest intensity of the emotion “happy” was expressed by judges with white and brown mushroom samples fermented with the *Lp. plantarum* No. 135 strain (on average, 0.013). The LAB strain used for fermentation and the interactions of the LAB strain used for fermentation and mushroom variety were significant in the intensity of the emotion “happy” induced in judges by mushroom samples (*p* ≤ 0.001 and *p* = 0.002, respectively). Additionally, between the intensity of the emotion “happy” and volatile compounds 1-octen-3-ol and 3-octanone, positive moderate correlations were found (r = 0.620, *p* ≤ 0.001 and r = 0.484, *p* = 0.007, respectively). Additionally, between the intensity of the emotion “happy” and the samples’ pH, a positive weak correlation was established (r = 0.398, *p* = 0.029). 

The LAB strain used for fermentation and the interactions of the LAB strain used for fermentation and mushroom variety were significant in the intensity of the emotion “sad” induced in judges by mushroom samples (*p* ≤ 0.001), and the highest expression of the emotion “sad” was found with *Lp. plantarum* No. 135 strain-fermented brown-variety mushroom samples and with *P. acidilactici* No. 29 strain-fermented white-variety mushroom samples (on average, 0.093). Positive weak and moderate correlations between the intensity of the emotion “sad” and the volatile compounds acetoin and 2,3-butanediol were found (r = 0.387, *p* = 0.035 and r = 0.514, *p* = 0.004, respectively). Additionally, negative correlations between the intensity of the emotion “sad” and benzaldehyde; 1-octen-3-ol; 3-octanone; nonanal; dodecane; and tetradecane were established (r = −0.610, *p* ≤ 0.001; r = −0.568, *p* ≤ 0.001; r = −0.377, *p* = 0.040; r = −0.364, *p* = 0.048; r = −0.520, *p* = 0.003; and r = −0.391, *p* = 0.033, respectively). 

All the analysed factors and their interactions were significant in the intensity of the emotion “angry” induced for judges by mushroom samples (*p* ≤ 0.001), and the lowest expression of the emotion “angry” was found with white-variety mushroom sample groups fermented with *Lc. casei* No. 210, *Lc. paracasei* No. 244, and *P. acidilactici* No. 29 strains. Positive correlations between the expression of the emotion “angry” intensity and the volatile compounds benzaldehyde; benzeneacetaldehyde; nonanal; benzo-2,3-pyrrole; and tetradecane were found (r = 0.370, *p* = 0.044; r = 0.587, *p* ≤ 0.001; r = 0.398, *p* = 0.029; r = 0.602, *p* ≤ 0.001; and r = 0.454, *p* = 0.012, respectively). 

The highest intensity of the emotions “surprised” and “scared” were found with brown-variety mushroom sample groups fermented with *P. acidilactici* No. 29 strain. Correlations between the expression of the emotion “surprised“ intensity and the analysed physicochemical and microbiological parameters of the mushroom samples were not found. However, between the intensity of the emotion “scared” and some volatile compounds (2,3-butanediol; dodecane; benzo-2,3-pyrrole; and 2,4-bis(1,1-dimethylethyl)phenol), TTA, and tyramine content, significant correlations were established (r = 0.362, *p* = 0.050; r = −0.476, *p* = 0.008; r = 0.641, *p* ≤ 0.001; r = −0.475, *p* = 0.008; r = 0.426, *p* = 0.019; and r = 0.694, *p* ≤ 0.001, respectively). Additionally, the interaction of the analysed factors was significant in the intensity of the emotion “scared” induced in judges by mushroom samples (*p* = 0.016). 

All the analysed factors and their interactions were significant in the intensity of the emotion “disgusted” induced for judges by mushroom samples (mushroom variety *p* = 0.013; LAB strain used for fermentation *p* = 0.001; factors interaction *p* ≤ 0.001), and the highest expression of the emotion “disgusted” was found with white-variety mushroom sample groups fermented with the *Lc. paracasei* No. 244 strain. Despite that the analysed factors were not significant in the intensity of emotion “contempt” induced in judges by mushroom samples, between the expression of the emotion “contempt” and some volatile compounds (2,3-butanediol; ethylhexanol; benzyl alcohol), TTA, and tyramine content, significant correlations were established (r = 0.504, *p* = 0.005; r = −0.554, *p* = 0.002; r = 0.454, *p* = 0.012; r = 0.424, *p* = 0.019; r = 0.575, *p* ≤ 0.001, respectively). 

Finally, most of the tested samples’ valences were negative, except for the non-fermented white mushroom variety and *P. acidilactici* No. 29 strain-fermented brown mushroom samples, and the LAB strain used for fermentation and the analysed factors’ interactions were significant on the valence value (*p* ≤ 0.001). 

Usually, mushrooms are incorporated into other food products, and their sensory profile and acceptability are evaluated [94]. There are only a few studies on the sensory analysis of fermented mushrooms. Liu et al. [50] reported that edible oyster mushrooms fermented with *Lactiplantibacillus pentosus* for 18 days received scores of 7–8 for overall acceptability. In the study of Jabłońska-Ryś et al. [19], 7 days of fermentation with *Lp. plantarum* 299v and *Lp. plantarum* EK3 was applied to *A. bisporus* and the scores of overall acceptability were lower compared to those obtained in our study. The evaluation of food-elicited emotions is an expanding field of interest in the food sector, as the emotional profile can be employed to differentiate foods with identical hedonic scores and plays an important role in estimating consumer purchase intentions [95,96]. Mushroom-elicited emotions were evaluated in the study of Tepsongkroh et al. [96], who found that sensory ratings and emotional response, measured with the Essense Profile^®^, of extruded snacks were notably affected when mushrooms *Volvariella volvacea* and *Pleurotus pulmonarius* were incorporated into the snack formula.

## 4. Conclusions

Fermentation with No. 210, No. 135, No. 244, and No. 29 strains induced changes in the physical properties of *A. bisporus* as well as volatile, BA, FA, and elicited emotion profiles. Fermented mushrooms had a darker colour and a higher count of viable LAB, but similar yeast and fungi counts compared to non-fermented ones. The total BA content in fermented *A. bisporus* was below the maximum level suggested for foods, and in most cases, fermented samples showed a higher content of polyunsaturated FA. The main volatile compounds in mushroom samples were 1-octen-3-ol and benzyl alcohol. The overall acceptability was similar for both non-fermented and fermented mushrooms. The LAB strain used for fermentation and the *A. bisporus* variety were significant factors in most of the elicited emotion intensities in the judges. This research provided beneficial information that can be further applied to the industrial production of fermented mushrooms. Furthermore, a wider spectrum of LAB for *A. bisporus* fermentation can be studied in the future to recommend the safest and most acceptable product for the consumer as well as the most appropriate LAB strain for the mushroom fermentation industry.

## Figures and Tables

**Table 1:** Colour characteristics and acidity parameters of the non-fermented and fermented mushroom samples.

Mushroom Samples	L*	a*	b*	pH	TTA, °N
	NBS				
Wnon-f	76.7 ± 1.25 j	6.28 ± 0.59 e	18.5 ± 0.09 g	6.80 ± 0.01 i	0.10 ± 0.03 a
Bnon-f	65.4 ± 1.14 i	7.90 ± 0.63 f	17.9 ± 0.11 f	6.66 ± 0.03 h	0.10 ± 0.02 a
W210	55.3 ± 0.98 h	3.47 ± 0.28 b	17.8 ± 0.13 f	4.90 ± 0.02 c	0.40 ± 0.03 d
B210	33.0 ± 0.29 b	4.88 ± 0.24 c	9.36 ± 0.08 a	5.44 ± 0.03 f	0.20 ± 0.01 b
W135	48.2 ± 0.47 f	3.06 ± 0.21 a,b	15.2 ± 0.10 e	5.33 ± 0.01 e	0.30 ± 0.02 c
B135	39.6 ± 0.32 c	6.07 ± 0.19 e	12.6 ± 0.08 c	5.72 ± 0.02 g	0.20 ± 0.01 b
W244	51.2 ± 0.49 g	3.13 ± 0.18 b	17.9 ± 0.09 f	4.24 ± 0.01 a	0.40 ± 0.03 d
B244	32.1 ± 0.31 a	5.43 ± 0.14 d	9.84 ± 0.06 b	4.33 ± 0.03 b	0.20 ± 0.01 b
W29	45.7 ± 0.43 e	2.83 ± 0.11 a	12.9 ± 0.09 d	5.44 ± 0.01 f	0.20 ± 0.02 b
B29	43.7 ± 0.36 d	4.54 ± 0.23 c	12.8 ± 0.05 d	5.27 ± 0.02 d	0.30 ± 0.01 c

W—*A. bisporus* white variety; B—*A. bisporus* brown variety; non-f—non-fermented samples; 210—fermented with *Lc. casei* No. 210 strain; 135—fermented with *Lp. plantarum* No. 135 strain; 244—fermented with *Lc. paracasei* No. 244 strain; 29—fermented with *P. acidilactici* No. 29 strain; TTA—total titratable acidity; °N—Neiman degree; L* lightness; a* redness or −a* greenness; b* yellowness or −b* blueness; NBS—National Bureau of Standards units. Data expressed as mean values (*n* = 9) ± standard error (SE). a–j Mean values within the columns with different letters are significantly different (*p* ≤ 0.05).

**Table 2:** Lactic acid bacteria, yeast, and fungi counts (log_10_ CFU/g) in non-fermented and fermented mushroom samples.

Mushroom Samples	LAB Count, log_10_ CFU/g	Yeasts and Fungi Count, log_10_ CFU/g
Wnon-f	1.56 ± 0.16 a	1.32 ± 0.27 a
Bnon-f	1.48 ± 0.17 a	1.45 ± 0.18 a
W210	7.82 ± 0.22 d	1.14 ± 0.21 a
B210	7.79 ± 0.11 d	1.12 ± 0.29 a
W135	7.53 ± 0.15 c,d	1.19 ± 0.22 a
B135	7.41 ± 0.18 c	1.25 ± 0.23 a
W244	6.53 ± 0.22 b	1.36 ± 0.37 a
B244	6.49 ± 0.19 b	1.28 ± 0.14 a
W29	6.51 ± 0.14 b	1.47 ± 0.25 a
B29	6.47 ± 0.17 b	1.33 ± 0.14 a

W—*A. bisporus* white variety; B—*A. bisporus* brown variety; non-f—non-fermented samples; 210—fermented with *Lc. casei* No. 210 strain; 135—fermented with *Lp. plantarum* No. 135 strain; 244—fermented with *Lc. paracasei* No. 244 strain; 29—fermented with *P. acidilactici* No. 29 strain; LAB—lactic acid bacteria; CFU—colony forming units. Data expressed as mean values (*n* = 9) ± standard error (SE). a–d Mean values within the columns with different letters are significantly different (*p* ≤ 0.05).

**Table 3:** Biogenic amines concentration (mg/kg) in mushroom samples.

Mushroom Samples	Biogenic Amine, mg/kg								
	Tryp-Tamine	Phenyl-Ethylamine	Putrescine	Cada-Verine	Histamine	Tyramine	Spermi-Dine	Spermine	Total Content
Wnon-f	nd	nd	nd	nd	nd	nd	183.9 ± 9.32 a,b	nd	183.9
Bnon-f	nd	nd	nd	nd	nd	nd	181.2 ± 8.54 a	nd	181.2
W210	nd	nd	nd	nd	nd	75.1 ± 6.58 a	184.9 ± 6.98 a	nd	260.0
B210	nd	nd	nd	nd	nd	85.5 ± 7.89 a	203.3 ±10.5 b	nd	288.7
W135	nd	nd	nd	nd	nd	87.6 ± 7.74 a	180.0 ± 8.69 a	nd	267.6
B135	nd	nd	nd	nd	nd	nd	222.3 ± 8.56 b	nd	222.3
W244	nd	nd	nd	nd	nd	nd	187.1 ± 9.54 a,b	nd	187.1
B244	nd	nd	nd	nd	nd	nd	186.9 ± 7.54 a,b	nd	186.9
W29	nd	nd	nd	nd	nd	nd	181.6 ± 9.76 a	nd	181.6
B29	nd	nd	nd	nd	nd	nd	203.3 ± 11.35 b	nd	203.3

W—*A. bisporus* white variety; B—*A. bisporus* brown variety; non-f—non-fermented samples; 210—fermented with *Lc. casei* No. 210 strain; 135—fermented with *Lp. plantarum* No. 135 strain; 244—fermented with *Lc. paracasei* No. 244 strain; 29—fermented with *P. acidilactici* No. 29 strain; nd—not determined. Data expressed as mean values (*n* = 9) ± standard error (SE). a–b Mean values within the columns with different letters are significantly different (*p* ≤ 0.05).

**Table 4:** Fatty acid content (% of the total fat content) of the mushroom samples.

Fatty Acids	Wnon-f	Bnon-f	W210	B210	W135	B135	W244	B244	W29	B29
	Fatty Acid Content, % of the Total Fat Content									
C16:0	12.3 ± 0.03 b	12.3 ± 0.02 b	13.0 ± 0.02 f	12.4 ± 0.04 c	12.4 ± 0.03 c	11.0 ± 0.02 a	12.5 ± 0.03 d	12.3 ± 0.02 b	12.7 ± 0.03 e	12.4 ± 0.02 c
C16:1	nd	0.625 ± 0.005 c	0.205 ± 0.002 a	0.376 ± 0.004 b	nd	nd	nd	nd	nd	nd
C18:0	2.07 ± 0.05 b	3.12 ± 0.06 g	2.33 ± 0.03 d	2.70 ± 0.06 f	1.75 ± 0.04 a	2.58 ± 0.06 e	2.23 ± 0.04 c	2.17 ± 0.07 b,c	1.69 ± 0.02 a	2.76 ± 0.06 f
C18:1	9.99 ± 0.05 h	9.59 ± 0.07 g	7.88 ± 0.05 f	5.57 ± 0.04 b	10.6 ± 0.09 i	3.44 ± 0.05 a	6.46 ± 0.07 d	6.73 ± 0.06 e	10.1 ± 0.09 h	6.08 ± 0.05 c
C18:2	75.6 ± 0.31 c	72.2 ± 0.28 a	74.9 ± 0.33 b	76.9 ± 0.19 d	75.3 ± 0.24 b,c	80.6 ± 0.43 f	78.8 ± 0.29 e	78.9 ± 0.31 e	75.5 ± 0.42 b,c	78.8 ± 0.27 e
C20:0	nd	1.11 ± 0.03 b	0.830 ± 0.021 a	1.04 ± 0.05 b	nd	1.13 ± 0.04 b	nd	nd	nd	nd
C22:0	nd	1.13 ± 0.02 c	0.905 ± 0.011 a	1.05 ± 0.02 b	nd	1.23 ± 0.01 d	nd	nd	nd	nd
	Fatty acid profile (%)									
SFA	14.4 ± 0.04 b	17.6 ± 0.05 h	17.1 ± 0.03 f	17.2 ± 0.04 g	14.2 ± 0.01 a	15.9 ± 0.03 e	14.7 ± 0.04 c	14.4 ± 0.06 b	14.4 ± 0.04 b	15.1 ± 0.03 d
MUFA	9.99 ± 0.07 e	10.2 ± 0.08 f	8.09 ± 0.07 d	5.94 ± 0.11 b	10.6 ± 0.09 g	3.44 ± 0.12 a	6.46 ± 0.14 c	6.73 ± 0.18 c	10.1 ± 0.10 e,f	6.08 ± 0.05 b
PUFA	75.6 ± 0.25 c	72.2 ± 0.32 a	74.9 ± 0.41 b	76.9 ± 0.38 d	75.3 ± 0.29 b,c	80.6 ± 0.52 f	78.8 ± 0.47 e	78.9 ± 0.33 e	75.5 ± 0.48 b,c	78.8 ± 0.36 e
Omega-3	nd	nd	nd	nd	nd	nd	nd	nd	nd	nd
Omega-6	75.6 ± 0.48 b	72.2 ± 0.31 a	74.9 ± 0.47 b	76.9 ± 0.44 c	75.3 ± 0.38 b	80.6 ± 0.51 e	78.8 ± 0.39 d	78.9 ± 0.61 d	75.5 ± 0.52 b	78.8 ± 0.54 d
Omega-9	9.99 ± 0.08 e	10.2 ± 0.10 f	8.09 ± 0.08 d	5.94 ± 0.15 b	10.6 ± 0.09 g	3.44 ± 0.14 a	6.46 ± 0.23 c	6.73 ± 0.11 c	10.1 ± 0.09 e,f	6.08 ± 0.18 b,c

W—*A. bisporus* white variety; B—*A. bisporus* brown variety; non-f—non-fermented samples; 210—fermented with *Lc. casei* No. 210 strain; 135—fermented with *Lp. plantarum* No. 135 strain; 244—fermented with *Lc. paracasei* No. 244 strain; 29—fermented with *P. acidilactici* No. 29 strain; C16:0—Palmitic acid; C16:1—Palmitoleic acid; C18:0—Stearic acid; C18:1—9-Octadecenoic acid; C18:2—Linoleic acid; C20:0—eicosanoic acid; C22:0—Docosanoic acid; SFA—saturated fatty acids; MUFA—monounsaturated fatty acids; PUFA—polyunsaturated fatty acids; nd—not determined. Data expressed as mean values (*n* = 9) ± standard error (SE). a–i Mean values within the lines with different letters are significantly different (*p* ≤ 0.05).

**Table 5:** Volatile compounds of the mushroom samples (% of the total volatile compounds).

RT, min	Volatile Compounds	Mushroom Samples									
		Wnon-f	Bnon-f	W210	B210	W135	B135	W244	B244	W29	B29
4.41	Acetoin	nd	nd	nd	5.42 ± 0.48 a	13.5 ± 1.14 b	23.5 ± 2.22 c	nd	nd	nd	nd
6.39	2,3-Butanediol	nd	nd	nd	22.4 ± 2.25 b	32.2 ± 3.29 c	17.6 ± 0.16 a	nd	nd	36.8 ± 2.41 c	nd
11.78	Benzaldehyde	7.40 ± 0.41 e	9.57 ± 0.52 f	3.65 ± 0.34 d	1.97 ± 0.15 b	0.377 ± 0.041 a	2.46 ± 0.25 c	3.77 ± 0.38 d	6.70 ± 0.54 e	nd	4.14 ± 0.35 d
12.43	1-Octen-3-ol	33.6 ± 2.95 g	20.8 ± 1.95 f	0.819 ± 0.045 a	2.89 ± 0.28 c,d	nd	3.20 ± 0.27 d	1.31 ± 0.12 b	2.50 ± 0.22 c	1.22 ± 0.11 b	4.19 ± 0.36 e
12.66	3-Octanone	7.55 ± 0.53 f	7.88 ± 0.47 f	0.819 ± 0.056 b	2.37 ± 0.25 d	0.253 ± 0.018 a	5.10 ± 0.49 e	1.16 ± 0.15 c	2.20 ± 0.21 d	2.25 ± 0.23 d	8.27 ± 0.41 f
12.96	2-Ethylhexanol	1.01 ± 0.10 a	2.27 ± 0.23 b	nd	0.871 ± 0.058 a	nd	1.89 ± 0.17 b	nd	nd	nd	nd
14.19	Benzyl alcohol	46.1 ± 3.44 b	50.4 ± 4.39 b	85.0 ± 5.32 d	50.5 ± 4.11 b	33.5 ± 2.98 a	31.5 ± 2.72 a	81.6 ± 6.35 d	67.6 ± 4.74 c	50.3 ± 4.11 b	71.2 ± 5.69 c,d
14.51	Benzeneacetaldehyde	1.47 ± 0.13 b	5.79 ± 0.52 e	1.88 ± 0.19 c	7.23 ± 0.61 f	0.782 ± 0.048 a	4.21 ± 0.36 d	nd	8.44 ± 0.53 g	1.31 ± 0.10 b	6.82 ± 0.52 e,f
16.44	Nonanal	0.491 ± 0.036 b	0.397 ± 0.035 a	2.00 ± 0.19 f	1.01 ± 0.09 d	0.788 ± 0.069 c	1.54 ± 0.14 e	2.31 ± 0.22 f	1.40 ± 0.13 e	1.16 ± 0.11 d	0.729 ± 0.041 c
19.35	Dodecane	0.835 ± 0.071 a	0.721 ± 0.069 a	2.23 ± 0.21 d	1.76 ± 0.16 c	1.12 ± 0.11 b	4.07 ± 0.28 f	3.65 ± 0.34 e,f	4.13 ± 0.39 f	3.09 ± 0.25 e	2.04 ± 0.19 c,d
22.04	Benzo-2,3-pyrrole	nd	nd	nd	nd	15.5 ± 0.16 c	1.32 ± 0.12 b	nd	0.535 ± 0.043 a	nd	nd
24.84	Tetradecane	0.732 ± 0.069 b	0.599 ± 0.041 a	2.40 ± 0.23 e	1.60 ± 0.15 d	0.977 ± 0.085 c	2.88 ± 0.25 f	3.71 ± 0.31 g	4.01 ± 0.35 g	3.10 ± 0.26 f	1.47 ± 0.15 c
27.74	2,4-bis(1,1-dimethylethyl)phenol	0.828 ± 0.073 b	1.56 ± 0.14 d	1.21 ± 0.11 c	1.97 ± 0.15 e	1.09 ± 0.08 c	0.694 ± 0.052 a	2.46 ± 0.22 f	2.54 ± 0.25 f	0.770 ± 0.058 a,b	1.12 ± 0.11 c

RT—retention time; W—*A. bisporus* white variety; B—*A. bisporus* brown variety; non-f—non-fermented samples; 210—fermented with *Lc. casei* No. 210 strain; 135—fermented with *Lp. plantarum* No. 135 strain; 244—fermented with *Lc. paracasei* No. 244 strain; 29—fermented with *P. acidilactici* No. 29 strain; RT—retention time; nd—not determined. Data expressed as mean values (*n* = 9) ± standard error (SE). a–g Mean values within the lines with different letters are significantly different (*p* ≤ 0.05).

**Table 6:** Overall acceptability and emotions induced in judges by mushroom samples.

Mushroom Samples	OA	Emotions Induced for Judges by Mushroom Samples (from 0 to 1)								
		Neutral	Happy	Sad	Angry	Surprised	Scared	Disgusted	Contempt	Valence
Wnon-f	8.05 ± 1.48 a	0.768 ± 0.039 a	0.120 ± 0.011 e	0.009 ± 0.002 a	0.007 ± 0.001 b	0.003 ± 0.001 a	nd	0.020 ± 0.004 a,b	0.001 ± 0.0005 a	0.088 ± 0.024 d
Bnon-f	7.42 ± 1.35 a	0.769 ± 0.061 a	0.037 ± 0.004 c	0.034 ± 0.004 b	0.011 ± 0.002 b,c	0.005 ± 0.002 b	0.001 ± 0.0003 a	0.065 ± 0.009 d	0.001 ± 0.0006 a	−0.065 ± 0.014 b
W210	6.06 ± 1.15 a	0.763 ± 0.048 a	0.022 ± 0.002 b	0.055 ± 0.004 c,d	0.004 ± 0.001 a	0.004 ± 0.001 b	0.001 ± 0.0004 a	0.051 ± 0.007 d	0.001 ± 0.0004 a	−0.068 ± 0.021 b
B210	5.89 ± 1.48 a	0.760 ± 0.065 a	0.038 ± 0.003 c	0.064 ± 0.005 d	0.007 ± 0.002 b	0.009 ± 0.003 b	0.001 ± 0.0002 a	0.039 ± 0.004 c	0.006 ± 0.0007 b	−0.055 ± 0.009 b
W135	5.25 ± 1.32 a	0.768 ± 0.074 a	0.011 ± 0.002 a	0.057 ± 0.006 c,d	0.015 ± 0.003 c	0.009 ± 0.004 b	0.002 ± 0.0005 b	0.026 ± 0.003 b	0.002 ± 0.0008 a	−0.077 ± 0.010 b
B135	5.29 ± 1.21 a	0.712 ± 0.069 a	0.014 ± 0.003 a	0.091 ± 0.008 e	0.007 ± 0.002 b	0.003 ± 0.001 a	nd	0.056 ± 0.005 d	0.001 ± 0.0005 a	−0.119 ± 0.014 a
W244	6.69 ± 0.96 a	0.718 ± 0.065 a	0.028 ± 0.004 b	0.063 ± 0.007 d	0.003 ± 0.001 a	0.002 ± 0.001 a	nd	0.084 ± 0.007 e	0.002 ± 0.0009 a	−0.096 ± 0.009 b
B244	6.87 ± 1.02 a	0.770 ± 0.072 a	0.021 ± 0.003 b	0.048 ± 0.005 c	0.016 ± 0.002 c	0.007 ± 0.002 b	0.001 ± 0.0004 a	0.015 ± 0.003 a	0.002 ± 0.0008 a	−0.048 ± 0.005 b
W29	5.88 ± 0.49 a	0.699 ± 0.059 a	0.028 ± 0.004 b	0.094 ± 0.007 e	0.004 ± 0.001 a	0.008 ± 0.003 b	0.003 ± 0.001 b	0.047 ± 0.005 c,d	0.002 ± 0.0006 a	−0.106 ± 0.012 a
B29	5.69 ± 0.65 a	0.666 ± 0.063 a	0.097 ± 0.005 d	0.057 ± 0.006 c,d	0.011 ± 0.002 b,c	0.035 ± 0.005 c	0.007 ± 0.002 c	0.016 ± 0.002 a	0.001 ± 0.0005 a	0.021 ± 0.005 c

W—*A. bisporus* white variety; B—*A. bisporus* brown variety; non-f—non-fermented samples; 210—fermented with *Lc. casei* No. 210 strain; 135—fermented with *Lp. plantarum* No. 135 strain; 244—fermented with *Lc. paracasei* No. 244 strain; 29—fermented with *P. acidilactici* No. 29 strain; OA—overall acceptability; nd—not determined. Data expressed as mean values (*n* = 10) ± standard error (SE). a–e Mean values within the columns with different letters are significantly different (*p* ≤ 0.05).

## Data Availability

The data used to support the findings of this study can be made available by the corresponding author upon request.
